# Influenza A Virus and *Mycoplasma pneumoniae* Coinfection Mediates Immune Dysregulation and Exacerbates Disease Severity

**DOI:** 10.3390/ijms262110487

**Published:** 2025-10-28

**Authors:** Yanan Wu, Qi Zhu, Jing Liu, Hongyun Chuan, Lingyu He, Mingqing Wang, Lilan Xu, Runfang Zhang, Yao Liu, Guoyang Liao, Weidong Li, Chengquan Sun, Jian Zhou

**Affiliations:** 1 Key Laboratory of Vaccine Research and Development for Major Infectious Diseases in Yunnan Province, Institute of Medical Biology, Chinese Academy of Medical Sciences, Peking Union Medical College, Kunming 650118, China; 2 Department of Industrial Transformation, Institute of Medical Biology, Chinese Academy of Medical Sciences and Peking Union Medical College, Kunming 650118, China; 3 Production Department, Institute of Medical Biology, Chinese Academy of Medical Sciences and Peking Union Medical College, Kunming 650118, China; 4 Scientific Research Office, Institute of Medical Biology, Chinese Academy of Medical Sciences, Peking Union Medical College, Kunming 650118, China

**Keywords:** coinfection, *Mycoplasma pneumoniae*, influenza, mouse model

## Abstract

Coinfection with influenza virus and *Mycoplasma pneumoniae* (MP) increases mortality during influenza pandemics; however, its specific impact on *Mycoplasma Pneumoniae* Pneumonia (MPP) patients or animal models remains unclear. The underlying mechanisms of *influenza A virus* (IAV)–MP interactions are not fully understood. To investigate the causes of heightened mortality, we established a lethal sequential coinfection mouse model using H3N2 influenza and MP. Coinfection led to prolonged viral persistence, enhanced pulmonary immune cell infiltration, and significantly elevated levels of inflammatory cytokines (IL-6, CCL3, CCL4, and G-CSF; *p* < 0.05–0.001), culminating in severe pneumonia. Notably, coinfected mice exhibited impaired CD8+ T-cell responses (*p* < 0.05) and increased pulmonary IL-6 and IL-1α levels (*p* < 0.05) compared with the controls. Our findings demonstrate that IAV-MP coinfection induces immune-mediated lung injury, which likely contributes to the observed mortality. This study provides critical insights into the immunopathogenesis of coinfection and suggests potential therapeutic targets for managing coinfected patients.

## 1. Introduction

The devastating impact of influenza-bacterial coinfections on pandemic mortality was first recognized during the 1918 influenza pandemic, where secondary bacterial infections were responsible for the majority of deaths [1]. This lethal synergy was confirmed during subsequent pandemics, including those caused by the H2N2 virus in 1957 and the H3N2 virus in 1968, establishing bacterial coinfection as a hallmark of severe influenza outcomes [1]. While conventional respiratory pathogens, such as *Streptococcus pneumoniae, Staphylococcus aureus,* and *Haemophilus influenzae*, have been extensively studied in this context [2,3], *Mycoplasma pneumoniae* (MP) stands out among atypical bacterial pathogens due to its high prevalence and distinct pathobiology. Furthermore, experimental studies by Bezuglova et al. demonstrated that MP-influenza coinfection in Syrian hamsters could even induce lung tumorigenesis [4]. The emerging threat of concurrent respiratory virus outbreaks—particularly the ongoing coronavirus pandemic—has renewed interest in understanding complex pathogen interactions. Recent studies on hospitalized children with *Mycoplasma pneumoniae* pneumonia (MPP) in China have indicated that coinfection with influenza virus accounts for 11.68–16.9% of all respiratory viral coinfections in MPP cases, representing a significant proportion. In some regions, such as southern Guangdong, the influenza virus has been identified as the most prevalent coinfecting pathogen in children with MPP. This viral coinfection has been shown to significantly aggravate clinical severity, prolong the treatment duration, and substantially increase the risk of refractory MPP and pulmonary complications [5,6]. Additionally, clinical evidence has confirmed that H3N2 is the predominant subtype involved in influenza virus and *Mycoplasma pneumoniae* coinfections, revealing a specific immunologic synergy and synchronized epidemic dynamics between them, which consequently leads to higher clinical morbidity [7]. It is for these reasons that the present study focused on H3N2 and *Mycoplasma pneumoniae* coinfection as the research subject. Despite these findings, research on MP and other atypical bacteria (e.g., Chlamydia pneumoniae) in influenza coinfections remains disproportionately limited compared with studies of conventional bacterial pathogens. Mounting evidence from animal models highlights MP’s role in exacerbating influenza pathogenesis. Studies in a bovine model coinfected with influenza D virus (IDV) and Mycoplasma bovis have demonstrated that this coinfection results in a synergistic amplification of both respiratory and systemic symptoms [8]. The coinfection of influenza virus and *Mycoplasma pneumoniae* exhibits a significant synergistic pathogenic effect. The underlying mechanism involves the influenza virus impairing host immune responses and disrupting mucosal barriers, thereby creating favorable conditions for *Mycoplasma pneumoniae* infection and ultimately leading to exacerbated lung injury and disease severity [9,10]. These findings support the hypothesis that MP acts as a critical “facilitator” of severe influenza outcomes. Clinically, influenza-MP coinfections are increasingly associated with more severe disease manifestations in humans [11,12,13]. However, the precise immunopathological mechanisms underlying this synergistic virulence remain poorly understood. Elucidating these mechanisms is crucial for improving the management of coinfected patients, particularly during overlapping seasonal epidemics of influenza and atypical bacterial infections.

## 2. Results

### 2.1. MP Infection Impairs Host Defense Against Influenza Virus

**Figure 1:**
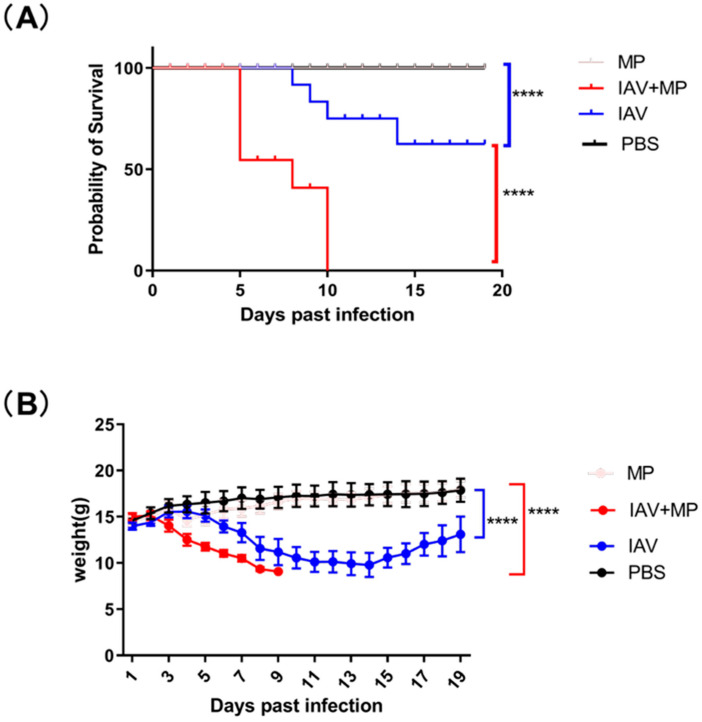
Survival and body weight of infected mice over 19 days. (**A**) Kaplan–Meier survival curves were generated for infected mice and compared using the log-rank test, which revealed a significant difference (**** *p* < 0.0001; n = 8 per group). (**B**) Changes in body weight were monitored over the 19-day period and analyzed using the log-rank test, demonstrating a significant effect (**** *p* < 0.0001; n = 8 per group).

To evaluate the impact of coinfection with influenza virus and *Mycoplasma pneumoniae* (MP) on disease severity, mice (n = 8 per group) were intranasally inoculated with either 50 μL of TCID50/mL pandemic H3N2 IAV strain, 5 × 10^7^ CFU of *Mycoplasma pneumoniae* FH strain, both pathogens simultaneously, or PBS as a control. While PBS-treated mice maintained normal body weight and showed 100% survival throughout the 19-day observation period, distinct disease patterns emerged in the infected groups (Figure 1).

Mice infected with IAV alone exhibited progressive weight loss beginning at 3 days post-infection (dpi), reaching a maximum average reduction of 4.21 g (30.1% of initial body weight, *p* < 0.0001) before showing signs of recovery by 14 dpi. The MP-only infected group showed no significant clinical symptoms or weight changes compared with the controls, though pathological examination revealed lung damage.

Strikingly, coinfected mice demonstrated rapid disease progression, with weight loss commencing at 3 dpi and peaking at approximately 5.94 g (39.6% of initial body weight, *p* < 0.0001). This group experienced dramatically reduced survival, with 37.5% mortality by 5 dpi (*p* < 0.0001 vs. single infection groups) and 100% mortality within 7 days post-MP inoculation. These findings demonstrate that IAV-MP coinfection synergistically enhances disease severity, leading to significantly worse outcomes than single pathogen infections, as evidenced by both accelerated mortality rates and exacerbated weight loss.

### 2.2. Immune Inflammation in Lung Injury Following Influenza Virus and Mycoplasma pneumoniae Coinfection

**Figure 2:**
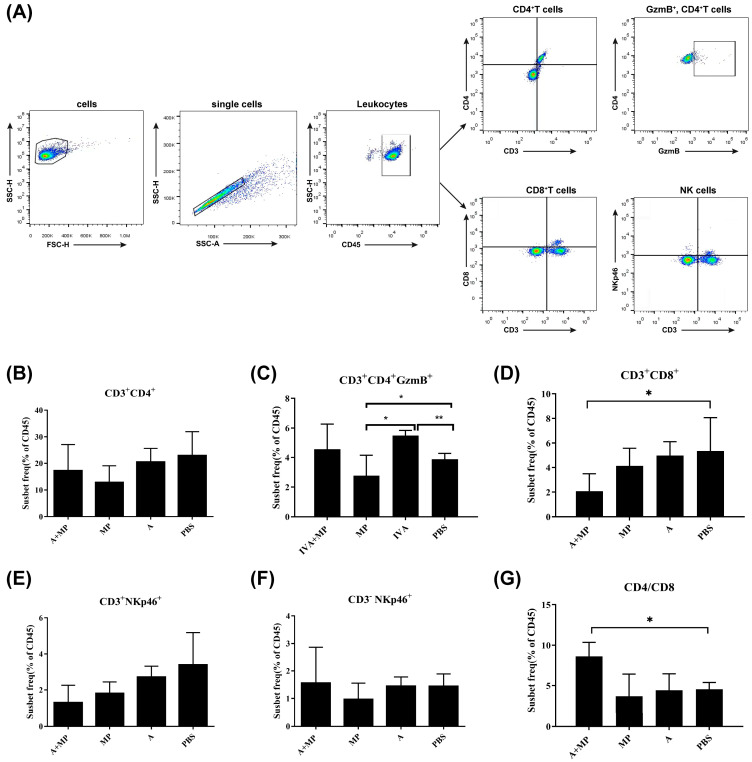
Flow cytometry strategy for analyzing surface marker expression in splenic immune cells. (**A**) Gating strategy for the identification of immune cell populations. (**B**–**D**,**G**) Statistical analysis of T cell populations. (**E**) Statistical analysis of NKT cells. (**F**) Statistical analysis of NK cells. Mice were coinfected with IAV and MP, infected with IAV alone, infected with MP alone, or treated with PBS as a control (n = 4 per group). Statistical significance is indicated as follows: * *p* < 0.05; ** *p* < 0.01.

Excessive inflammatory responses are known to contribute to severe pathological outcomes during infections with either influenza A virus (IAV) or *Mycoplasma pneumoniae* (MP) [14,15]. To investigate the inflammatory profiles associated with these infections, we performed flow cytometric analysis of splenic immune cell populations from mice subjected to either single or coinfections (Figure 2).

Compared with phosphate-buffered saline (PBS)-treated control mice, most infected groups exhibited a reduction in CD8^+^CD3^+^ T cells. Notably, mice coinfected with IAV and MP demonstrated a significantly greater decrease in CD8^+^CD3^+^ T cells than either of the single-infection groups (*p* < 0.05). However, no significant differences were observed in the numbers of CD4^+^ T cells or total CD3^+^ T cells among the coinfected, IAV-infected, and MP-infected groups. As a result, the CD4/CD8 ratio was significantly elevated in coinfected mice relative to single-infection and control groups (*p* < 0.05).

Interestingly, the frequency of GMB^+^CD4^+^CD3^+^ cells—indicative of cytotoxic CD4^+^ T-cell activity—was significantly increased in IAV-infected mice compared with both the coinfected and control groups (*p* < 0.01). In contrast, MP-infected mice exhibited significantly lower levels of GMB^+^CD4^+^CD3^+^ cells relative to the IAV-infected and control mice (*p* < 0.05), suggesting that MP infection may impair the cytotoxic T lymphocyte (CTL) response following IAV infection, potentially contributing to MP-induced immunosuppression.

**Figure 3:**
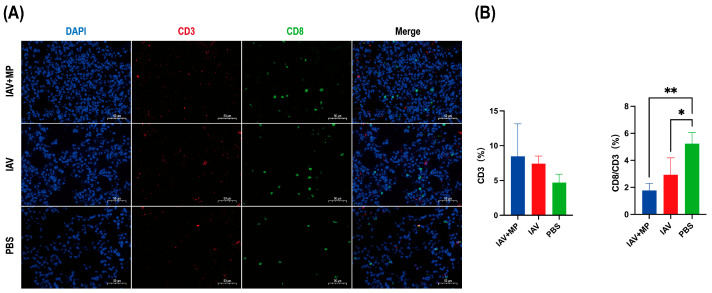
Immunofluorescence analysis of T-cell infiltration in lung tissues from infected mice. Lung specimens were collected at 24 h post-infection and subjected to immunofluorescence staining for (**A**) CD3 (red) and CD8 (green). (**B**) Quantitative analysis of the percentage of CD3^+^ T cells and the ratio of CD3^+^CD8^+^ cells to total CD3^+^ cells was performed using Aipathwell v2 software. Scale bar: 50 µm (n = 4 per group). * *p* < 0.05, ** *p* < 0.01 versus control group.

Immunofluorescence staining of lung sections obtained 24 h post-infection was performed to evaluate T-cell responses (Figure 3). Immunofluorescence analysis of the lung sections revealed that IAV-MP coinfection induced a discernible upward trend in total CD3^+^ T-cell infiltration compared with the PBS control. However, this was accompanied by a significant alteration in T-cell composition. Specifically, the proportion of CD8^+^ cytotoxic T cells within the CD3^+^ population was significantly reduced in both the IAV single-infection and coinfection groups (*p* < 0.05 and *p* < 0.01, respectively). Although the difference between the two infected groups was not statistically significant, the reduction was more pronounced following coinfection, suggesting an exacerbation of CD8^+^ T-cell loss or impaired recruitment that may contribute to heightened disease severity.

**Figure 4:**
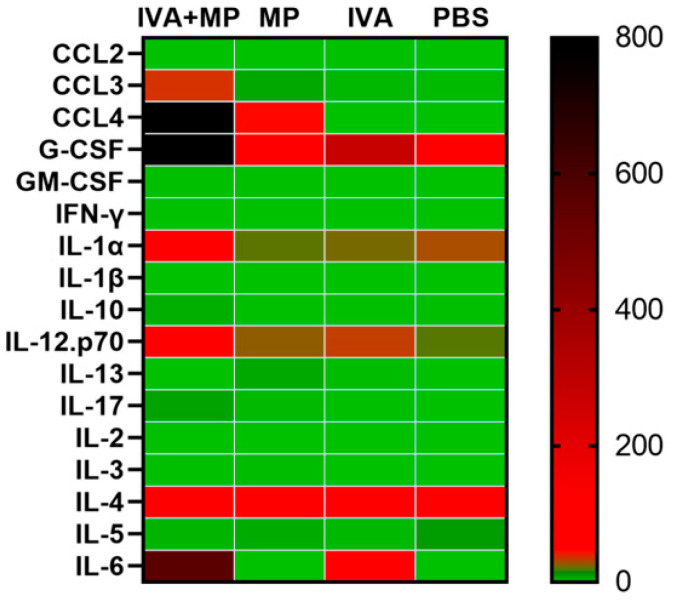
Heatmap of 17 inflammatory cytokines expressed in coinfected and singly infected mice. The expression levels of 17 inflammatory cytokines were compared between the coinfected mice and singly infected mice (n = 4 per group).

**Figure 5:**
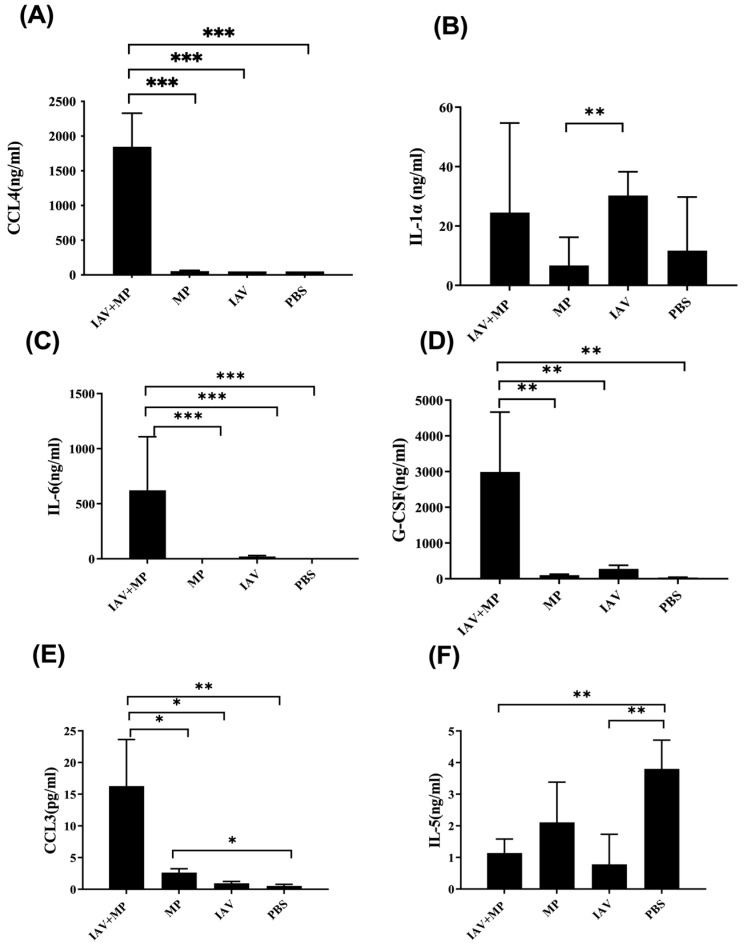
Differential expression of chemokines and cytokines in plasma from coinfected mice. (**A-F**) Plasma concentrations of (**A**) CCL4, (**B**) IL-1α, (**C**) IL-6, (**D**) G-CSF, (**E**) CCL3, and (**F**) IL-5. Mice were coinfected with IAV and MP, infected with IAV alone, infected with MP alone, or mock-treated with PBS (n = 4 per group) Statistical significance is indicated as * *p* < 0.05, ** *p* < 0.01, *** *p* < 0.001.

We further assessed systemic inflammation by measuring the plasma levels of 17 inflammatory cytokines on day 4 post-infection (Figure 4). Coinfected mice displayed significantly elevated levels of IL-6, CCL4, CCL3, G-CSF, and IL-1α, while the IL-5 levels were markedly reduced compared with the controls (*p* < 0.01). These cytokine shifts likely reflect the heightened disease severity observed in coinfected animals (Figure 5).

**Figure 6:**
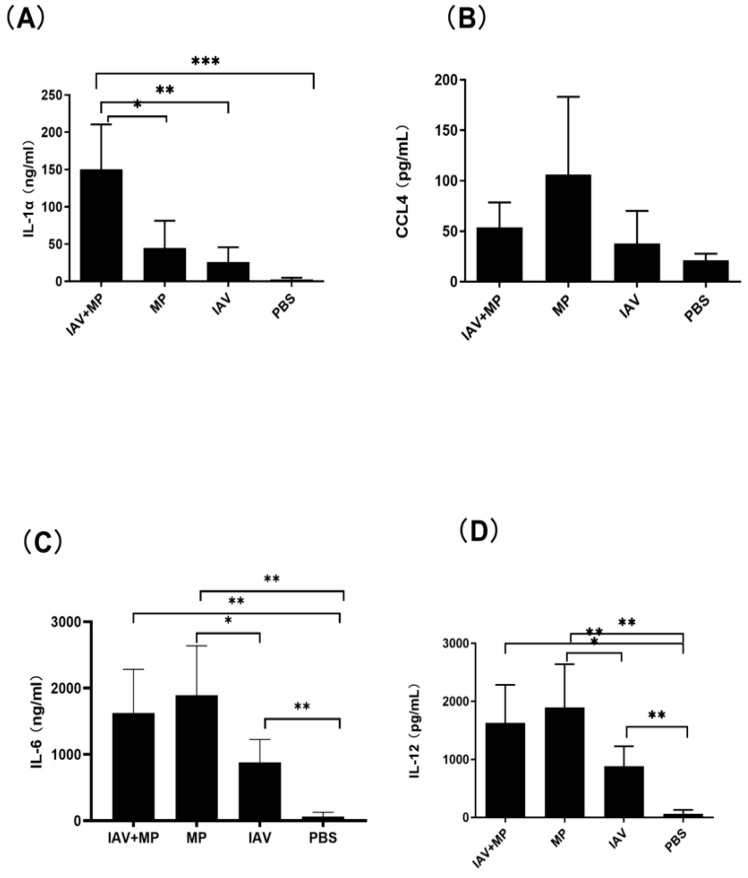
Pulmonary inflammatory response following coinfection. (**A**–**D**) Concentrations of (**A**) IL-1α, (**B**) CCL4, (**C**) IL-6, and (**D**) IL-12 in lung tissue homogenates. Mice were coinfected with IAV and MP, infected with IAV alone, infected with MP alone, or mock-treated with PBS (n = 4 per group). Statistical significance is indicated as * *p* < 0.05, ** *p* < 0.01, *** *p* < 0.001.

Consistent with these findings, ELISA measurements of lung homogenates revealed significantly higher levels of IL-6, CCL4, IL-1α, and IL-12 in coinfected mice (*p* < 0.05–0.001; Figure 6). Interestingly, while CCL4 was notably increased in coinfected lungs, MP-only infected mice exhibited the highest absolute levels of CCL4. No significant differences were observed in the lung levels of CCL2, GM-CSF, IL-10, IL-1β, IL-13, IL-17, IL-2, IL-3, IL-4, IL-8, or IFN-γ among the coinfected and single-infection groups.

**Figure 7:**
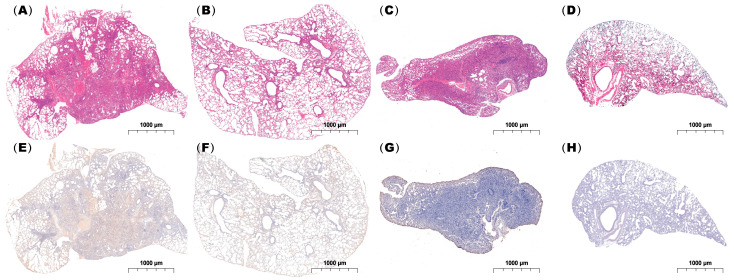
Histopathological analysis of lung tissues from infected mice. Lung specimens were collected at 24 h post-infection and subjected to (**A**–**D**) H&E staining and (**E**–**H**) immunohistochemical staining using an IAV-NP-specific antibody. (**A**,**E**), (**B**,**F**), (**C**,**G**), and (**D**,**H**) show mice coinfected with IAV followed by MP, infected with IAV alone, infected with IAV alone, and administered PBS as a control, respectively. Scale bar: 1 mm (n = 3 per group).

Histopathological examination demonstrated that both single and coinfected mice developed bronchiolitis and varying degrees of alveolitis, with viral antigens detected in the bronchial epithelium. However, lungs from the coinfected mice exhibited more extensive immune cell infiltration and pronounced pathological alterations including severe alveolar damage. Inflammatory exudates rich in immune cells and erythrocytes nearly obliterated the alveolar airspaces in coinfected animals (Figure 7), in contrast to the moderate pneumonia observed in singly infected mice.

Collectively, these results indicate that coinfection with IAV and MP leads to enhanced immune cell infiltration, dysregulated cytokine production, and more severe lung pathology compared with single infections. These outcomes are likely driven by synergistic interactions between IAV and MP, amplifying inflammatory responses and contributing to the observed immunopathology.

## 3. Discussion

Coinfection with viral and bacterial pathogens is increasingly recognized as a common occurrence in nature [16]. This study aimed to investigate the impact of coinfection with influenza virus (IAV) and *Mycoplasma pneumoniae* (MP). Coinfection generally exacerbates mortality, and we established a lethal coinfection model in which mice were sequentially infected with IAV followed by MP, mimicking the clinical scenario where secondary MP infection after influenza leads to increased mortality. Our study demonstrates that coinfection with IAV and MP led to significantly higher mortality, exacerbated lung pathology, an enhanced inflammatory cytokine response, and a reduction in CD8^+^ T cells compared with either single infection. These findings indicate a severe dysregulation of the host response during coinfection, culminating in synergistic disease severity.

The data presented in this study highlight the pivotal role of severe pneumonia in the death of mice coinfected with IAV and MP. IAV-infected mice exhibited continuous and severe body weight loss until day 6 post-infection (dpi) with a 50% survival rate. In contrast, the lethal coinfection group showed rapid body weight loss starting at 3 dpi, and this group experienced 100% mortality by 9 dpi. These findings suggest that coinfection with IAV and MP results in significant short-term survival impairment, likely due to severe bronchopneumonia and massive lung hemorrhage. An overactive host immune response appears to be a key factor in determining lung pathology in coinfected mice, with IAV infection potentially facilitating the growth of bacteria.

Furthermore, coinfection with influenza A virus and *Mycoplasma pneumoniae* is associated with an increased incidence of severe disease and serious pulmonary complications, significantly worsening the clinical condition of children with MPP [17,18]. Coinfections of viral and bacterial pathogens are common in nature, and the symptoms of SARS-CoV-2, IAV, and MP infections often overlap including fever, cough, pneumonia, and acute respiratory distress syndrome [19,20,21]. Our histopathological findings, which demonstrated more extensive immune cell infiltration and notable pathology such as severe alveolar damage in the lungs of coinfected mice, are in line with existing research.

The exacerbated lung pathology and increased mortality we observed were closely linked to a sharp rise in pro-inflammatory cytokines. This suggests that coinfection triggers a dysregulated and potentially detrimental host immune response. In our mouse model, serum levels of IL-6, CCL4, CCL3, G-CSF, IL-1α, and IL-5 were positively correlated with disease severity (coinfection > IAV-only > MP-only). Strong inflammatory cytokine and chemokine responses are associated with the high morbidity and mortality observed during pathogenic viral infections [22,23]. The lung, as the site of infection, showed consistent increases in IL-1α and IL-6, paralleling serum responses in coinfected mice.

The spleen plays a crucial role in CD4^+^ and CD8^+^ T-cell development. Notably, secondary MP infection reduced the number of cytotoxic CD4^+^ T cells in the spleen following primary IAV(H3N2) infection. This reduction in cytotoxic CD4^+^ T cells may be directly related to the immune response against the pathogens [24]. Cytotoxic CD4^+^ T cells are important for antiviral and antitumor immunity and contribute to inflammation. Additionally, previous studies have shown that CD8^+^ T cells activated during IAV infection can acquire cytolytic activity, which plays a protective role against lethal challenges [25,26,27]. In our study, the coinfection group exhibited a significant decrease in CD8+ T cells in the spleen compared with both the IAV-only and MP-only groups. Meanwhile, the number of CD8 T cells during IAV-MP coinfection in lung tissue was significantly reduced, and the decrease in CD8 T cells may be due to lethal challenges. Previous clinical studies have demonstrated that a decreased proportion of CD8^+^ T cells is closely associated with the progression to pneumonia in children with IAV and MP coinfection, a finding that is consistent with our results [28].

Few studies have explored the critical role of MP in IAV infections. Atypical bacterial infections are considered potential risk factors for severe influenza complications. In this study, we found that coinfection with IAV and MP significantly increased the levels of cytokines and inflammatory chemokines and the inflammation in the lung. Dysregulation of cytokine release due to these pathogens is associated with severe lung infections and increased mortality. More importantly, this study revealed that IAV superimposed with MP infection would decrease the number of CD8 T cells in the lung and spleen tissues.

A limitation of this study is that it primarily focused on coinfection-related mortality due to severe pneumonia but did not clarify the mechanisms of pneumonia development during coinfection. Further research is needed to investigate the underlying mechanisms. Nevertheless, our data suggest that atypical bacterial coinfection with IAV significantly increases mortality and warrants further attention and study.

## 4. Methods and Materials

### 4.1. Ethics Statement

All experimental animal procedures were approved by the Committee on Live Animal Care and Research at the Institute of Medical Biology, Chinese Academy of Medical Sciences and Peking Union Medical College, with approval number [DWSP202408012]. Cervical dislocation served as the approved method of euthanasia. Additionally, 10-day-old embryonated chicken eggs (Beijing Vital River Laboratory Animal Technology Co., Ltd., Beijing, China) were inoculated with influenza A virus for viral stock production.

### 4.2. Mice, Influenza Virus, and Mycoplasma pneumoniae

Female wild-type BALB/c mice (4–6 weeks old) were procured from Charles River Laboratories (Wilmington, MA, USA).

The influenza A virus strain *A/Fujian/Tongan/196/2009 (H3N2)* was propagated in the allantoic cavities of 9–11-day-old embryonated specific-pathogen-free (SPF) chicken eggs (Beijing Vital River Laboratory Animal Technology Co., Ltd., Beijing, China) at 37 °C for 48–72 h. Viral particles were subsequently purified through sucrose density gradient ultracentrifugation (20–60% *w*/*v*) at 100,000× *g* for 2 h at 4 °C, following the established protocols. Viral titers were quantified using two complementary methods: (1) assessment of cytopathic effects (CPE) in MDCK cells infected with tenfold serial dilutions of viral stocks, and (2) a hemagglutination (HA) assay with 1% guinea pig erythrocytes. The median tissue culture infectious dose (TCID50) was calculated using the Reed–Muench endpoint dilution method, with the results expressed as log10 TCID50/mL.

The *Mycoplasma pneumoniae* FH reference strain (ATCC 15531; American Type Culture Collection, Rockville, MD, USA) was cultured aseptically in pleuropneumonia-like organism (PPLO) broth (BD Biosciences, Sparks, MD, USA) supplemented with 10% heat-inactivated horse serum (Gibco, Thermo Fisher Scientific, Auckland, New Zealand) at 37 °C in a 5% CO_2_ atmosphere until the mid-logarithmic phase. For the preparation of infectious stocks, cultures were harvested by centrifugation at 12,000× *g* for 15 min at 4 °C. The pelleted bacteria were washed three times with sterile phosphate-buffered saline (PBS, pH 7.4) and resuspended in fresh PBS. Bacterial viability was assessed by enumerating colony forming units (CFUs) on PPLO agar plates containing 10% horse serum. Briefly, serial tenfold dilutions (10^−1^ to 10^−8^) of bacterial suspensions were plated in triplicate and incubated at 37 °C with 5% CO_2_ for 5–7 days until visible colonies appeared. CFU counts were determined using a Sterile culture dish. (Corning, Oneonta, NY, USA) and expressed as CFU/mL.

### 4.3. Mouse Model of Coinfection with Influenza A Virus (IAV) and Mycoplasma pneumoniae (MP)

All mice were maintained in BSL-2, specific-pathogen-free (SPF) facilities. Mice were randomly assigned to four groups (n = 8 per group): coinfection group (IAV + MP), IAV single-infection group (influenza A virus only), MP single-infection group (*Mycoplasma pneumoniae* only), and control group (PBS-treated, non-infected).

For the coinfection group, mice were intranasally inoculated with 100 TCID50 of IAV H3N2 strain, resuspended in 50 μL sterile PBS on day 0. After a 48-h interval, the mice were sequentially intranasally inoculated with 5 × 10^7^ CFU of *Mycoplasma pneumoniae* FH strain, resuspended in 50 μL of sterile PBS for two consecutive days (days 2–3). In the IAV single-infection group, mice were administered 100 TCID50 of the IAV H3N2 strain intranasally on day 0, followed by intranasal PBS administration (50 μL) on days 2–3.For the MP single-infection group, mice received intranasal PBS (50 μL) on day 0, followed by infection with 5 × 10^7^ CFU of the *Mycoplasma pneumoniae* FH strain on days 2–3. Control animals were treated with intranasal PBS (50 μL) on days 0–3 following the same administration schedule as experimental groups.

### 4.4. Antibodies and Flow Cytometric Analysis

Spleen-derived cells were washed and incubated with monoclonal antibodies specific for mouse CD45 (clone 30-F11), CD3 (clones 17A2 and 145-2C11), CD4 (clone GK1.5), CD8 (clone 53-6.7), CD56 (clone 29A1.4), and GzmB (clone NGZB), all of which were purchased from Invitrogen. The cell concentration in each group was determined using a hemocytometer. For intracellular staining, cells were fixed and permeabilized using Intracellular Staining Perm Wash buffer and Fixation buffer ((BioLegend, San Diego, CA, USA), following the manufacturer’s protocols. After the final wash step, the samples were resuspended in 100 μL of PBS. Data acquisition was performed using the Cytor FL EX flow cytometer (Beckman Coulter, Indianapolis, IN, USA), and subsequent analysis was conducted with NovoExpress software v.1.2.4.

### 4.5. Measurement of Serum Cytokine

For in vivo cytokine assessment, blood samples were collected from each mouse and centrifuged at 1000× *g* for 10 min at 4 °C to obtain serum. The supernatant was aliquoted and stored at −80 °C until further analysis. Cytokine concentrations were quantified using a Luminex 200 system (Luminex Corporation, Austin, TX, USA) with a multiplex bead-based immunoassay, following the manufacturer’s protocol. The following cytokines were analyzed: CCL2, CCL3, CCL4, G-CSF, GM-CSF, IFN-γ, IL-1α, IL-1β, IL-2, IL-3, IL-4, IL-5, IL-6, IL-10, IL-12p70, IL-13, and IL-17. Data are presented as mean values from four biologically independent mice per group and visualized as a heatmap using Microsoft Excel.

### 4.6. Lung Cytokine

Lung tissue homogenates were centrifuged at 1000× *g* for 10 min at 4 °C to pellet cellular debris. The supernatants were collected, and cytokine levels (IL-6, IL-12, CCL4, and IL-1α) were quantified using commercially available ELISA kits (eBioscience, Thermo Fisher Scientific, Shanghai, China) according to the manufacturer’s instructions. Absorbance was measured with a Bio-Rad iMark™ (Bio-Rad Laboratories, Hercules, CA, USA)Microplate Absorbance Reader, and cytokine concentrations were determined from standard curves and expressed as picograms per milliliter (pg/mL). All samples were assayed in duplicate, and the results were averaged.

### 4.7. Hematoxylin and Eosin (H&E) Staining and Immunohistochemistry

Lung tissues were fixed overnight in 4% paraformaldehyde (PFA) in phosphate-buffered saline (PBS) at 4 °C, dehydrated, and embedded in paraffin. Serial sections (5 μm thick) were cut and stained with hematoxylin and eosin (H&E) according to standard protocols.

For immunohistochemical detection of the IAV H3N2 strain, sections were incubated with a primary anti-mouse influenza A virus nucleoprotein (NP) monoclonal antibody (clone 3C8; Novoprotein, Suzhou, China), followed by a horseradish peroxidase (HRP)-conjugated goat anti-mouse IgG (H + L) secondary antibody (Servicebio, Wuhan, China). Diaminobenzidine (DAB) was used as the chromogen, and the sections were counterstained with hematoxylin for nuclear visualization.

### 4.8. Immunofluorescence Staining

Lung tissues were fixed in 4% PFA, paraffin-embedded, and sectioned at 4 μm. Following deparaffinization and antigen retrieval in citrate buffer (pH 6.0) via a 90 °C water bath, sequential TSA-based multiplex immunofluorescence staining was conducted. Sections were first incubated with rabbit anti-CD3 (1:2000; SinoBiological, Beijing, China), followed by an HRP-conjugated goat anti-rabbit secondary antibody and signal amplification with Cy3-conjugated tyramide (TSA 555, Servicebio, Wuhan, China). HRP activity was then inactivated by microwave heating of the sections, which were placed in a container filled with citrate buffer (pH 6.0). Subsequently, the process was repeated for rabbit anti-CD8 (1:1000; Servicebio, Wuhan, China) using the same secondary antibody and IF-488 conjugated tyramide (TSA 488,Servicebio, Wuhan, China). Nuclei were counterstained with DAPI, and images were captured using a Nikon Eclipse C1 upright fluorescence microscope and analyzed with Aipathwell v2 software.

### 4.9. Data Analysis

Data are expressed as the mean ± standard deviation (SD). All statistical analyses were performed using Prism Software (version 9.5). The log-rank test was used for survival curve analysis, and two-way analysis of variance (ANOVA) with Bonferroni’s multiple comparison test was used to assess statistical significance between the experimental groups. Differences were considered statistically significant at *p* < 0.05.

## 5. Conclusions

In conclusion, our study establishes that sequential coinfection with IAV and *Mycoplasma pneumoniae* synergistically exacerbates disease severity in a murine model, resulting in significantly elevated mortality. This lethal outcome is driven by severe pulmonary pathology, a sharp pro-inflammatory cytokine response, and a coinfection-specific CD8^+^ T-cell lymphopenia, collectively indicating a critical failure of adaptive immunity that permits uncontrolled pathogen persistence and tissue injury. We will further investigate the specific mechanisms underlying these severe outcomes to inform future therapeutic strategies.

## Data Availability

The raw data supporting the conclusions of this article will be made available by the authors on request.
